# Dual Roles of *METCAM* in the Progression of Different Cancers

**DOI:** 10.1155/2012/853797

**Published:** 2012-03-28

**Authors:** Guang-Jer Wu

**Affiliations:** ^1^Department of Microbiology and Immunology, Emory University, School of Medicine, Atlanta, GA 30322, USA; ^2^Department of Bioscience Technology, Chung Yuan Christian University, Chung-Li 32023, Taiwan

## Abstract

*METCAM*, an integral membrane cell adhesion molecule (*CAM*) in the *Ig*-like gene superfamily, is capable of performing typical functions of *CAM*s, such as mediating cell-cell and cell-extracellular interactions, crosstalk with intracellular signaling pathways, and modulating social behaviors of cells. *METCAM* is expressed in about nine normal cells/tissues. Aberrant expression of *METCAM* has been associated with the progression of several epithelial tumors. Further *in vitro* and *in vivo* studies show that *METCAM* plays a dual role in the progression of different tumors. It can promote the malignant progression of several tumors. On the other hand, it can suppress the malignant progression of other tumors. We suggest that the role of *METCAM* in the progression of different cancer types may be modulated by different intrinsic factors present in different cancer cells and also in different stromal microenvironment. Many possible mechanisms mediated by this *CAM* during early tumor development and metastasis are suggested.

## 1. Introduction

**Figure 1:**
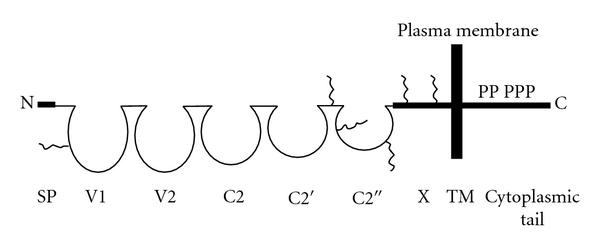
*HuMETCAM* protein structure. SP stands for signal peptide sequence: V1, V2, C2, C2′, and C2′′ for five Ig-like domains (each held by a disulfide bond) and X for one domain (without any disulfide bond) in the extracellular region, and TM for transmembrane domain. P stands for five potential phosphorylation sites (one for *PKA*, three for *PKC*, and one for *CK2*) in the cytoplasmic tail. The six conserved N-glycosylation sites are shown as wiggled lines in the extracellular domains of V1, the region between C2′ and C2′′, C2′′, and X.

Human *METCAM* (*huMETCAM*), a *CAM* in the immunoglobulin-like gene superfamily, is an integral membrane glycoprotein. Alternative names for *METCAM* are *MUC18* [1], *CD146* [2], *MCAM* [3], *MelCAM *[4], *A32 *[5], and *S-endo 1* [6]. To avoid confusion with mucins and to reflect its biological functions, we have renamed *MUC18* as *METCAM* (*met*astasis *CAM*), which means an immunoglobulin-like *CAM* that affects or regulates metastasis, [7]. The *huMETCAM *has 646 aminoacids that include a N-terminal extracellular domain of 558 aminoacids, which has 28 aminoacids characteristics of a signal peptide sequence at its N-terminus, a transmembrane domain of 24 aminoacids (amino acid number 559–583), and a cytoplasmic domain of 64 aminoacids at the C-terminus. *HuMETCAM *has eight putative N-glycosylation sites (*Asn-X-Ser/Thr*), of which six are conserved, and are heavily glycosylated and sialylated resulting in an apparent molecular weight of 113,000–150,000. The extracellular domain of the protein comprises five immunoglobulin-like domains (V-V-C2-C2-C2) [1, 7] and an X domain [7]. The cytoplasmic tail contains peptide sequences that will potentially be phosphorylated by protein kinase A (*PKA*), protein kinase C (*PKC*), and casein kinase 2 (*CK 2*) [1, 7, 8]. My lab has also cloned and sequenced the mouse *METCAM *(*moMETCAM*) cDNA, which contains 648 aminoacids with a 76.2% identity with *huMETCAM*, suggesting that *moMETCAM *is likely to have biochemical properties and biological functions similar to the human counterpart [9]. The structure of the *huMETCAM *protein is depicted in Figure 1, suggesting that *METCAM*, similar to most *CAMs,* plays an active role in mediating cell-cell and cell-extracellular interactions, crosstalk with many intracellular signaling pathways, and modulating the social behaviors of cells [7].

*HuMETCAM *is expressed in a limited number of normal tissues, such as hair follicular cells, smooth muscle cells, endothelial cells, cerebellum, normal mammary epithelial cells, basal cells of the lung, activated T cells, intermediate trophoblast [10], and normal nasopharyngeal epithelial cells [11]. The protein is overly expressed in most (67%) malignant melanoma cells [1], and in most (more than 80%) premalignant prostate epithelial cells (PIN), high-grade prostatic carcinoma cells, and metastatic lesions [12, 13]. *HuMETCAM* is also expressed in other cancers, such as gestational trophoblastic tumors, leiomyosarcoma, angiosarcoma, haemangioma, Kaposi's sarcoma, schwannoma, some lung squamous and small cell carcinomas, some breast cancer, some neuroblastoma [10], and also nasopharyngeal carcinoma [11] and ovarian cancer [14].

It is now well documented that in addition to tissue-specific signatures in different cancer types, cancers from different tissues also express some common genes [15–17]. One group of them is cell adhesion molecules (*CAMs*). *CAMs* do not merely act as a molecular glue to hold together homotypic cells in a specific tissue or to facilitate interactions of heterotypic cells; *CAMs *also actively govern the social behaviors of cells by affecting the adhesion status of cells and modulating cell signaling [18]. They control cell motility and invasiveness by mediating the remodeling of cytoskeleton [18]. They also actively mediate the cell-to-cell and cell-to-extracellular matrix interactions to allow cells to constantly respond to physiological fluctuations and to alter/remodel the surrounding microenvironment for survival [19]. They do so by crosstalk with cellular surface growth factor receptors, which interact with growth factors that may be secreted from stromal cells or released from circulation and embedded in the extracellular matrix [18, 19]. Thus, an altered expression of *CAMs* affects the motility and invasiveness of many tumor cells *in vitro* and metastasis *in vivo* [18, 19]. *CAMs *also play an important role in the favorable soil that provides a proper microenvironment at a suitable period to awaken the dormant metastatic tumor cells to enter into an aggressive growth phase. Actually, the metastatic potential of a tumor cell, as documented in many carcinomas, is the consequence of a complex participation of many over- and under-expressed *CAMs* [18, 19]. Based on the above information, aberrant expression of *huMETCAM *may also affect the motility and invasiveness of many tumor cells *in vitro* and metastasis *in vivo.* It is logical to hypothesize that *HuMETCAM/MUC18* should play an important role in promoting the malignant progression of many cancer types [7, 18]. However, recently we observed an unexpected opposite function of *METCAM/MUC18* in the malignant progression of a mouse melanoma subline and ovarian cancer cells, in which it functioned as a tumor and metastasis suppressor (Wu, unpublished results). In this paper, we will review its dual roles in the tumorigenesis and metastasis in different cancer types.

## 2. *METCAM* and Tumorigenesis

*METCAM*-induced tumorigenesis has been studied in melanoma, prostate cancer, breast cancer, and ovarian cancer. Overexpression of *METCAM* may have no effect, a negative effect, or a positive effect on tumorigenesis, dependent upon the cell lines used, as shown in the following.

### 2.1. *METCAM* and Melanoma Tumorigenesis

**Figure 2:**
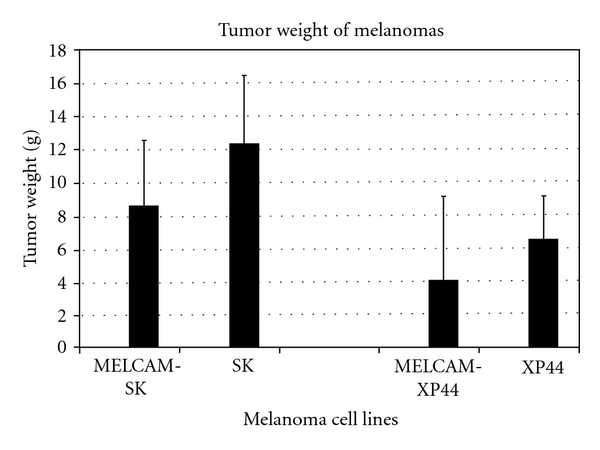
Effect of overexpression of *huMETCAM* on tumor formation of two human melanoma cell lines, *SK* and *XP-44* [20]. *MELCAM-SK* and *MELCAM-XP-44* were two clones of human melanoma cell lines, *SK* and *XP-44*, respectively, which were transfected with *huMETCAM* and expressed a high level of *huMETCAM*. Statistical analysis was not possible because detailed data was not provided.

**Figure 3:**
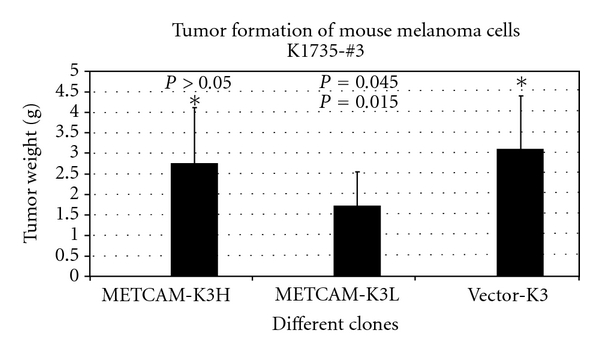
Effect of overexpression of *moMETCAM* on tumor formation of a mouse melanoma cell line *K1735* subline number 3 [21]. METCAM-K3H and METCAM-K3L were two K3 clones transfected with *moMETCAM* cDNA; expressed a high and a low level of *moMETCAM*, respectively. Vector-K3, as a negative control, was one clone transfected with an empty vector and did not express any *moMETCAM. *Asterisk was the reference for the *P* value calculation. The *P* values should be compared with the reference (asterisk) on the same row.

Overexpression of *METCAM* had a slight tumor suppression effect on tumorigenesis of human melanoma cells in xenograft mice [20], as shown in Figure 2, but it had no effect on tumorigenesis of two sublines, number 3 and number 10, of the mouse melanoma cell line K1735 in syngeneic mice [21].  Figure 3 shows only the effect of *moMETCAM *on the tumorigenesis of K1735-3.

**Figure 4:**
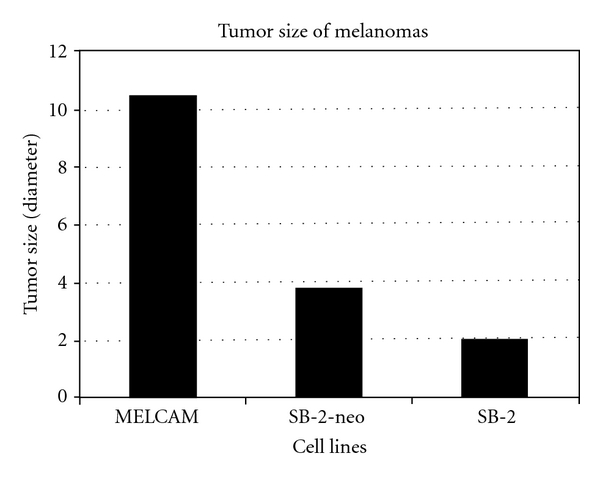
Effect of overexpression of *huMETCAM* on tumor formation of a human melanoma cell line *SB-2* [3]. *SB-2* is a human melanoma cell line, which did not express any *huMETCAM*. SB-2-neo is the SB-2 cells transected with the empty vector, as a negative control. *MELCAM* is a clone of the SB-2 cells which were transfected with *huMETCAM *cDNA and expressed a high level of *huMETCAM*. Since tumor formation was only shown in one nude mouse for each clone, statistical analysis was not possible.

Only one group showed that overexpression of *METCAM* increased tumorigenesis of a human melanoma cell line in xenograft mice [3]; however, the results were questionable because only the tumorigenicity of one mouse injected with *METCAM*-expressing clone and one mouse with control cells was determined, and thus no standard deviations were indicated and no statistical analysis was done, as shown in Figure 4.

**Figure 5:**
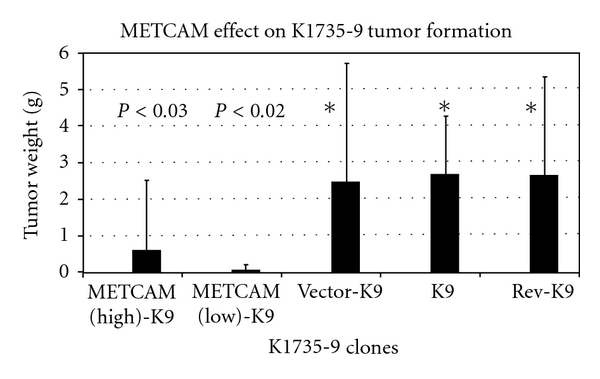
Effect of overexpression of *moMETCAM* on tumor formation of a mouse melanoma *K1735* subline number 9 (*K9*) in immune competent syngeneic *C3H* mice. METCAM-K9H and METCAM-K9L were two transfected clones, which expressed a high and a low level of *moMETCAM*, respectively. Vector-K9 was one clone transfected with the empty vector, as a negative control. K9 was parental K1735 subline number 9 cells, also as a negative control. Rev-K9, in which the *moMETCAM* cDNA was inserted into the expression vector in antisense orientation, is also as a control clone. Vector-K9, K9, and Rev-K9 did not express any *moMETCAM*.

The most compelling evidence for its tumor suppressor effect is in the subline number 9 of the mouse melanoma cell line *K1735* (*K9) *in syngeneic *C3H* mice. Overexpression of *moMETCAM *in the *K9* cells decreased subcutaneous tumorigenesis in immunocompetent syngeneic *C3H *mice [22, 23], as shown in Figure 5. 

### 2.2. *METCAM* and Breast Cancer Tumorigenesis

**Figure 6:**
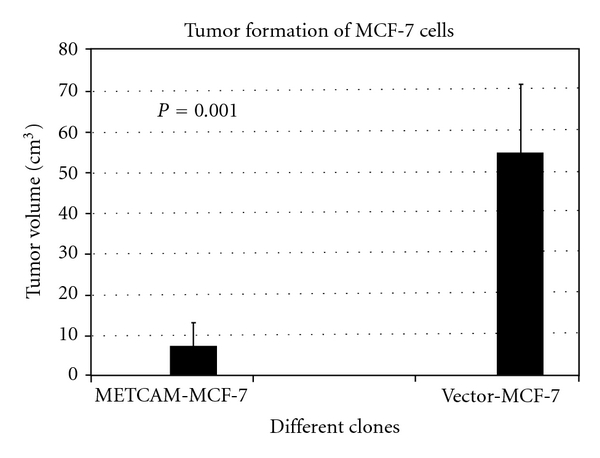
Effect of overexpression of *huMETCAM* on tumor formation of a human breast cancer cell line *MCF-7* [24]. METCAM-MCF-7 was a clone, which expressed a high level of *huMETCAM/MUC18* after transfection with the cDNA. Vector MCF-7 was a clone, which did not express any *huMETCAM *after transfection with an empty vector. Cells were injected subcutaneously into female *SCID *mice [24].

Shih et al. showed that *METCAM* was not expressed in *MCF-7* cell line [24], and they showed that the overexpression of *huMETCAM *in *MCF-7* cells suppressed tumor formation of the cells in *SCID* mice, as shown in Figure 6, suggesting that *METCAM *is a possible tumor suppressor in breast cancer [24].

**Figure 7:**
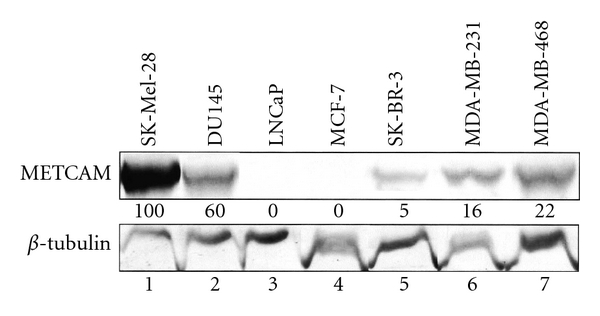
Expression of *huMETCAM* in four human breast cancer cell lines, *MCF-7*, *SK-BR-3*, *MDA-MB-231*, and *MDA-MB-468*. *SK-Mel-28*, a human melanoma cell line, which expressed a very high level of *huMETCAM*, was used as a positive control (100%). Two human prostate cancer cell lines, *DU145* and *LNCaP*, which expressed different levels of *huMETCAM* (60% and 0%, resp.) were used as positive and negative controls.

We have confirmed from their Western blot and immunohistochemistry results that *METCAM *is not expressed in *MCF-7* cells (0%), very weakly expressed in *SK-BR-3* cells (5%), and weakly expressed, though slightly higher levels than the above two cells lines, in the human mammary cancer cell lines, *MDA-MB-231* (a low metastatic cell line) (16%), and *MDA-MB-468* (a high metastatic cell line) (22%), as shown in Figure 7.

**Figure 8:**
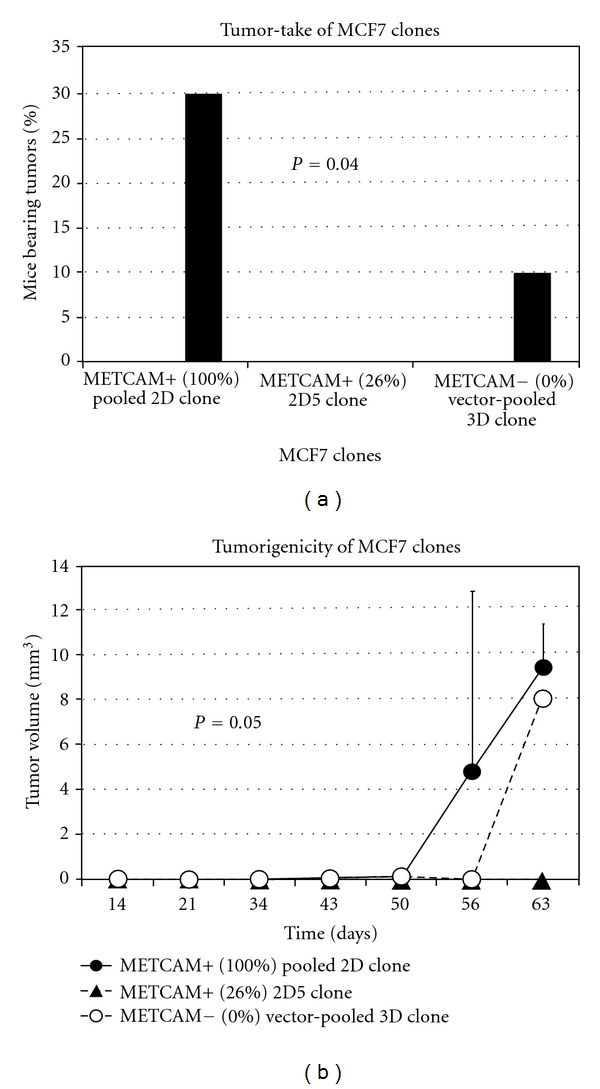
Effect of overexpression of *huMETCAM* on tumor-take (a) and tumorigenicity (b) of a human breast cancer cell line *MCF7 in female SCID mice*. METCAM+ clone 2D pooled was a pooled clone, which expressed 100% of *huMETCAM*, and METCAM+ clone 2D5, which expressed 26% of *huMETCAM*, after transfection with the *huMETCAM* cDNA. Vector clone 3D pooled was a pooled clone, which did not express any *huMETCAM*, after transfection with an empty vector. Cells were injected subcutaneously into female SCID mice [30].

**Figure 9:**
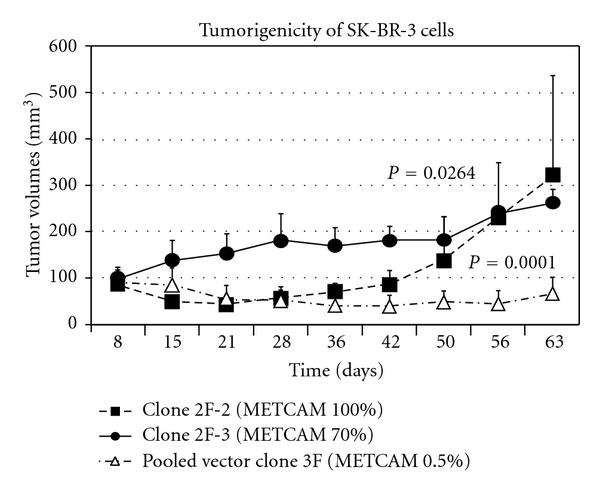
Effect of overexpression of *huMETCAM* on tumor formation of a human breast cancer cell line *SK-BR-3* in female nude mice [31]. Clone 2F-2 and clone 2F-3 expressed 100% and 70% of *METCAM*, respectively. Pooled vector clone 3F expressed only about 0.5% of *METCAM*, as a vector control.

Recently gene expression profiles of breast cancer cell lines have indicated that the gene expression profiles of *MCF-7* and *SK-BR3* are more closely related to the luminal subtype of the breast cancers, whereas those of *MDA-MB-231* and *MDA-MB-468* are more closely related to the basal-like subtype [25, 26]. It appeared that *METCAM *is not or weakly expressed in cell lines established from luminal subtypes, but it is moderately expressed in cell lines established from basal-like subtypes, *MDA-MB-231* and *MDA-MB-468*. Recently Ouhtit et al. [27] found that overexpression of *METCAM* inhibited the *in vitro* invasiveness of *MDA-MB-231* cells, supporting the notion of Shih et al. On the contrary, Garcia et al. [28] and Zabouo et al. [29] supported the opposite role of *METCAM *in the progression of human breast cancer cells in that it plays a role of tumor promoter. However, all three groups did not substantiate their claim with studies in animal models. To resolve this controversy, we recently reinvestigated the role of *METCAM* in the tumorigenesis of human breast cancer cells in animal models and found that overexpression of *METCAM* promoted the tumorigenesis of four human breast cancer cell lines, *MCF7*, *SK-BR-3*, *MDA-MB-231*, and *MDA-MB-468* [30, 31]. Tumorigenesis of *MCF-7* in female *SCID* mice [30] is shown in Figure 8, and that of *SK-BR-3* in female nude mice [31] in Figure 9.

Thus, the tumor suppression role of *METCAM* in tumorigenesis of human breast cancer cells is not supported by the above evidence. On the contrary, it suggests the alternative notion that *METCAM* increased tumorigenesis and perhaps also the metastasis of human breast cancer cells.

### 2.3. *METCAM* and Ovarian Cancer Tumorigenesis

**Figure 10:**
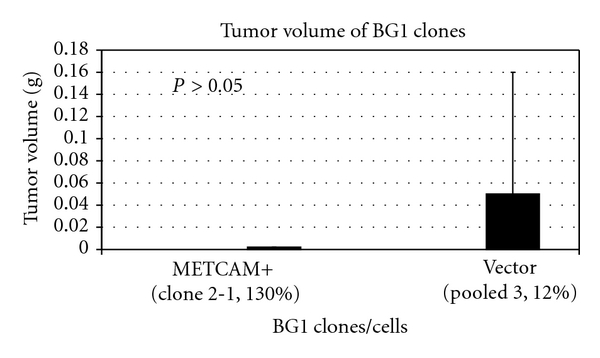
Effect of overexpression of *huMETCAM* on tumorigenicity of a human ovarian cancer cell line BG-1 in an orthotopic site (the intraperitoneal cavity) in female SCID mice. Clone 2-1 expressed 130% of *METCAM.* The vector control, pooled clone 3, expressed 12% of *METCAM*.

Recently, both our group and another group found that *METCAM* was upregulated in human ovarian cancer specimens, suggesting that *METCAM* may be a marker for the poor prognosis of ovarian cancer patients [14, 32], and that *METCAM* may play a positive role in the development of ovarian cancer [14, 32]. However, preliminary animal tests (injection of *BG-1* cells in nonorthotopic, subcutaneous sites of female nude mice) show that overexpression of *METCAM* did not have any significant effect on the tumor formation of a human ovarian cancer cell line, *BG-1* (data not shown). To rule out the possibility that this effect might be an artifact because the tests were carried out in the nonorthotopic, subcutaneous sites, which did not provide a proper microenvironment for tumorigenesis, we carried out further tests of the effect of overexpression of *METCAM* on tumorigenesis of *BG-1* cells by injecting the clones in an orthotopic site, the intraperitoneal cavity of female *SCID* mice. We found that tumorigenesis of BG-1 clones was also very poor, suggesting that estrogen supplement by subcutaneous implantation may enhance the tumorigenesis of *BG-1* cells in both immunodeficient mice. Nevertheless, the test of the effect of overexpression of *METCAM* on tumorigenesis of *BG-1* cells in orthotopic sites had a somewhat suppressive effect, as shown in Figure 10 [33].

**Figure 11:**
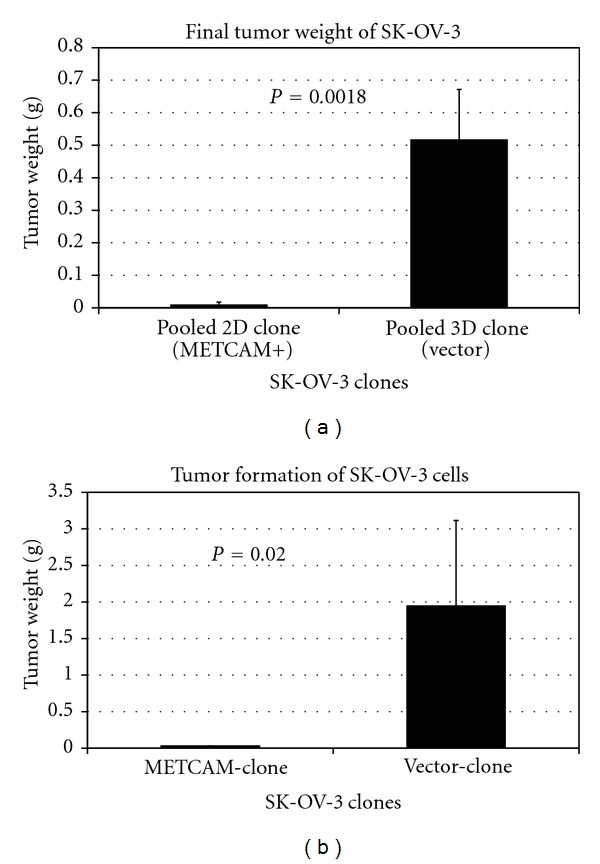
Effect of overexpression of *huMETCAM* on the final tumor weight at *S.C.* sites (a) and orthotopic sites (the intraperitoneal cavity) (b) of a human ovarian cancer cell line *SK-OV-3*. Both Pooled 2D clone and METCAM-clone expressed 100% of *METCAM*. Both pooled 3D clone (vector) and vector clone expressed 0.5% of *METCAM. *

We also carried out animal studies by using another human ovarian cancer cell line, *SK-OV-3 *and found that overexpression of *METCAM* suppressed tumorigenesis of *SK-OV-3* cells at both nonorthotopic, subcutaneous sites, as well as an orthotopic site (the intraperitoneal cavity) [34], as shown in Figure 11.

### 2.4. *METCAM* and Prostate Cancer Tumorigenesis

**Figure 12:**
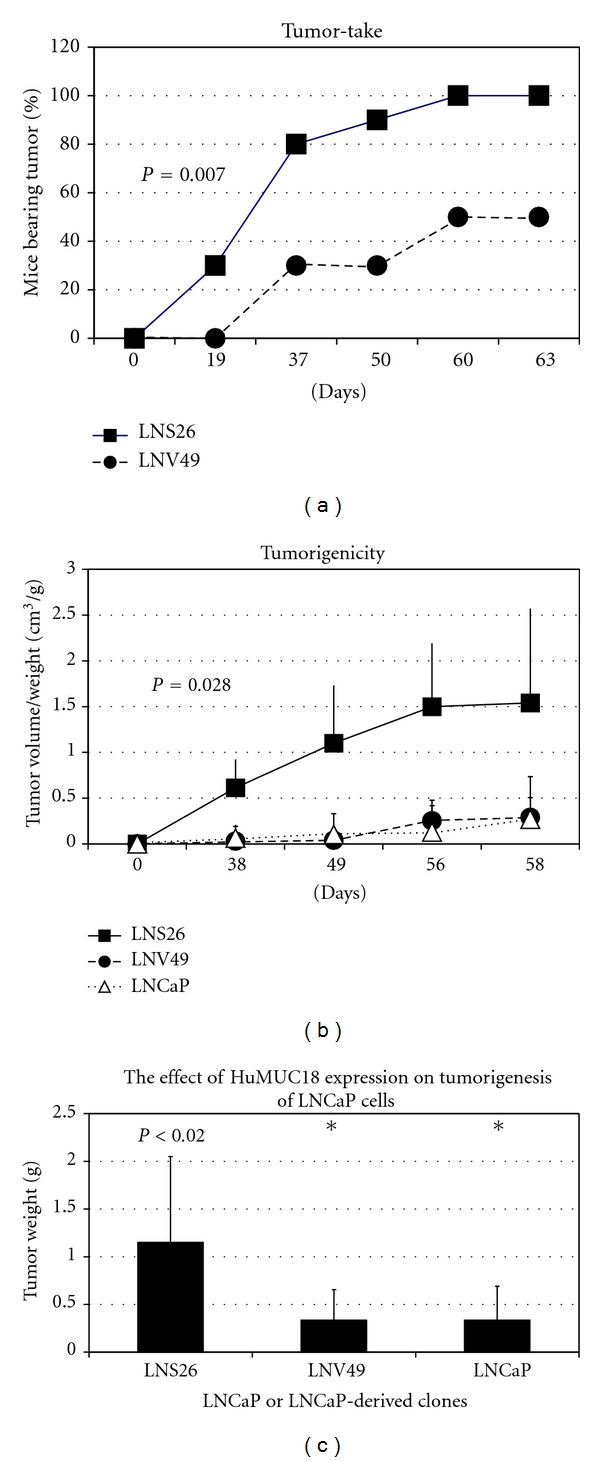
Enforced expression of *huMETCAM* in *LNCaP* cells resulted in an increased tumor-take (a), tumorigenicity (b), and final tumor weight (c) [35]. Clone LNS26 expressed *METCAM*. Both the vector control clone, LNV49, and the parental *LNCaP* cells did not express any *METCAM*.

Overexpression of *METCAM* significantly increases the tumor-take and promote tumorigenicity and tumorigenesis of a human prostate cancer cell line, *LNCaP,* as shown in Figure 12 [35, 36].

### 2.5. *METCAM* Tumorigenesis of Other Cancer Cell Lines

**Figure 13:**
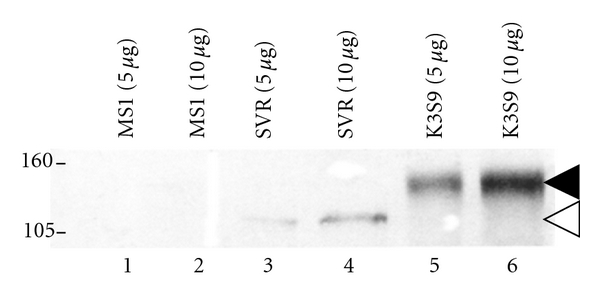
Expression of *moMETCAM* in mouse angiosarcoma cell lines [7]. *MS1* is an immortalized mouse endothelial cell line, which expressed a barely detectable (low) level of *moMETCAM* and was nontumorigenic. *SVR* is a mouse angiosarcoma cell line, which had been transfected with *H-Ras* gene, also expressed *moMETCAM*, and was tumorigenic. K3S9, a clone derived from mouse melanoma *K1735* subline number 3 which had been transfected with a *moMETCAM* cDNA gene, expressed a high level of *moMETCAM* and formed tumor efficiently in syngeneic mice (C3H). Western blot was carried out and detected by our chicken anti-*moMETCAM* antibody [9]. The smaller molecular weight of the *moMETCAM* (about 115 kDa) in angiosarcoma cell lines was probably due to less glycosylation than that in the mouse melanoma cell lines or in most human cancer cell lines (about 150 kDa).

We found that *moMETCAM* was expressed at a higher level in a mouse angiosarcoma clone, *SVR*, which was transfected with *H-Ras*, than in the immortalized normal endothelial cell line control, *MS-1 *(Figure 13). The higher level of *moMETCAM* expression appeared to correlate with the higher tumorigenicity of the *SVR* cell line [7, 37], suggesting a positive role for *METCAM* in promoting angiosarcoma [7].

There is a negative correlation of *METCAM* expression with the human nasopharyngeal carcinoma specimens, suggesting that *METCAM* may also play a tumor suppressor role in the tumorigenesis of nasopharyngeal carcinoma [11]. A tumor suppressor role of *METCAM* may also be implicated in haemangiomas, since *METCAM* expression was decreased during the progression of haemangiomas [38].

## 3. *METCAM* and Metastasis

*METCAM*-induced metastasis has been studied in melanoma, prostate cancer, osteosarcoma, and ovarian cancer lines. Overexpression of *METCAM* in melanoma cells mostly have a positive effect on the metastasis of human melanoma cell lines in immunodeficient mice (both athymic nude and *SCID* mice) [3, 20], mouse melanoma cell lines in syngeneic mice [7, 21], and a human prostate cancer cell line, *LNCaP*, in nude mice [7, 13, 36]. Overexpression of *METCAM* also has a positive effect on the metastasis of osteosarcoma cell lines [39]. Surprisingly, we have recently found that overexpression of *METCAM* has a negative effect on the metastasis of one subline, number 9, of mouse melanoma cell K1735 [22, 23] and ovarian cancer cell lines [30–32]. Details are described in the following.

### 3.1. *METCAM* and Melanoma Metastasis

*HuMETCAM* was originally found to be abundantly expressed on the cellular surface of most malignant human melanomas; since then, it has been postulated to play a role in the progression of human melanoma [1]. This notion is also supported by the positive correlation of *moMETCAM *expression with the metastatic ability of several mouse melanoma cells lines [9]. Definitive proof comes from the results that the stably ectopic expression of the *huMETCAM* cDNA gene in three nonmetastatic human cutaneous melanoma cell lines increases the metastatic abilities of these cell lines in immune-deficient mouse models [3, 20]. Furthermore, the stable, ectopic expression of *moMETCAM* cDNA in two low-metastatic mouse melanoma cell lines increases the metastatic abilities of these cell lines in immune-competent syngeneic mice [21]. However, *METCAM* enables melanoma cells to establish pulmonary metastasis only when the cells are injected into the tail vein (experimental metastasis assay) [3, 20, 21], thus bypassing the initial stages of metastasis. No metastasis was found when *METCAM*-expressing melanoma cells were injected subcutaneously (spontaneous metastasis assay) either in immune-deficient mouse models [3, 20] or in immune-competent syngeneic mouse models [21]. Taken together, *METCAM* definitely promotes the metastasis of melanoma cells, but at later stages [7]; thus overexpression of *METCAM* did not initiate the metastasis of melanoma cells. This result is consistent with the recent observation that fibroblast growth factor 2, but neither *huMETCAM* nor integrin actually initiates the malignant progression of subcutaneous melanocyte into melanoma [40].

**Figure 14:**
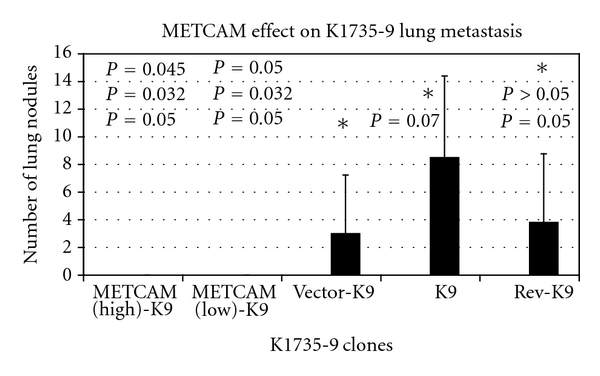
Enforced expression of *moMETCAM* suppressed lung nodule formation of mouse melanoma *K9* cells in syngeneic *C3H* mice. Clones METCAM (high)-K9 and METCAM (low)-K9 clones expressed high and low levels of *moMETCAM*, respectively. Vector-K9, K9 parental cells, and Rev-K9, in which the *moMETCAM* cDNA was inserted into the expression vector in antisense orientation, were the control clones that did not express any *moMETCAM*.

In contrast to these results, overexpression of *moMETCAM* in one mouse melanoma cell line *K1735 *subline number 9 (*K9*) decreased pulmonary lung nodule formation when cells were injected into tail veins (experimental metastasis test) [22, 23], as shown in Figure 14.

### 3.2. *METCAM* and Prostate Cancer Metastasis

Overexpression of *METCAM* is not limited to melanoma as previously thought [7, 10]. Our group has pioneered the successful determination of *huMETCAM* expression in prostate cancer cells and tissues using our chicken polyclonal *anti-huMETCAM* and carried out extensive studies of *huMETCAM*-mediated prostate cancer metastasis [8]. We have used molecular biological and immunological methods to study the expression of *huMETCAM* in three established prostate cancer cell lines and human prostate cancer tissues, and in immunohistochemical studies of paraffin-embedded human prostate cancer tissue sections [7, 8, 12, 13]. From the results, we have suggested that *huMETCAM *may be a new diagnostic marker for the metastatic potential of human prostate cancer. This is further corroborated by results of a positive correlation of *moMETCAM* expression with the progression of mouse prostate adenocarcinoma in a transgenic mouse model, *TRAMP *[41]. From these results, we have also suggested that *huMETCAM* may be a key determinant in promoting tumorigenesis and metastasis of human prostate cancer cells [7]. To test this hypothesis, we determined the effect of ectopic expression of *huMETCAM* on the ability of human prostate *LNCaP *cells to form tumor in the prostate gland and to initiate metastasis in nude mice. We found that overexpression of *METCAM *had a positive effect on the metastasis of the human prostate cancer cell, *LNCaP*, when the cells were injected at the orthotopic site (the dorsolateral lobes of the nude mice) [36]. The metastatic lesions were found in multiple organs, such as seminal vesicles, ureter, kidney, and periaortic lymph nodes [36]. Different mice had metastatic lesions in one or two organs, but all of them had metastatic lesions in the lymph nodes. The parental *LNCaP* cells, which do not express any *METCAM,* can form tumors in the prostate, but these tumors did not manifest any metastasis. The metastatic lesions in the bones were not examined. But our recent preliminary results appear to show that overexpression of *METCAM* may be able to enhance establishment of the growth of a bone-homing *C42B* clone of *LNCaP *cells in nude mice. Further tests are in process [Wu et al., unpublished results].

Taken together, *METCAM *can actually initiate the metastasis of *LNCaP* cells, thus affecting the progression of prostate cancer cells at the early stage of metastasis [7, 36].

### 3.3. *METCAM* and Osteosarcoma Metastasis

Recently, one group has shown that *METCAM* is overly expressed in two of the six human osteosarcoma cell lines. Overexpression of *METCAM *increased the spontaneous lung metastasis of an osteosarcoma cell line *KRIB*. The metastasis can be blocked by a humanized antibody against *METCAM,* suggesting *METCAM* plays a positive role in the progression of osteosarcomas [39].

### 3.4. *METCAM* and Ovarian Cancer Metastasis

**Figure 15:**
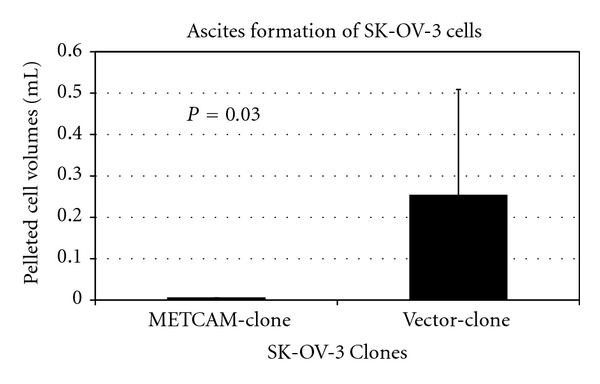
Enforced expression of *huMETCAM* in human ovarian *SK-OV-3* cells suppressed solid tumor formation and ascites formation in the intraperitoneal cavity. METCAM-clone expressed 100% of *METCAM*. Vector-clone expressed 0.5% of *METCAM*.

Recently we found that overexpression of METCAM/MUC18 suppressed metastasis and ascites formation of *SK-OV-3* cells in the intraperitoneal cavity [34], as shown in Figure 15.

### 3.5. *METCAM* and Metastasis of Other Cancer Cell Lines

Decreased expression of *METCAM* has been correlated with the progression of haemangioma, suggesting the possible negative effect of *METCAM* on progression of haemangioma [38]. Though *METCAM* was downregulated in nasopharyngeal carcinoma, interestingly it was upregulated again in metastatic lesions in nasopharyngeal patients, suggesting that *METCAM *may play a positive role in the malignant progression of nasopharyngeal carcinoma after a transient suppression of tumorigenesis [11].

 Taken together, we suggest that the possible tumor and metastasis suppressor role of *METCAM* may not be limited to melanoma and ovarian cancers, and that this may be a new function of *METCAM* yet to be explored.

**Summary 3.5**

Table 1 summarizes the possible role of *METCAM* in the tumorigenesis and metastasis of various tumors/cancers.Taken together, *huMETCAM* is a tumor promoter for prostate and breast cancers, and a metastatic gene for most melanoma cell lines, prostate cancer, osteosarcoma, and perhaps, breast cancer and nasopharyngeal carcinoma. It is a tumor suppressor for a mouse melanoma subline and ovarian cancers, and perhaps, haemangioma and nasopharyngeal carcinoma; it is a metastasis suppressor for a mouse melanoma subline, ovarian cancer, and perhaps, haemangioma.

## 4. Mechanisms of *METCAM*-Mediated Tumorignesis and Metastasis

 How does *METCAM* mediate or regulate tumorigenesis and metastasis of cancer cells? By deducing knowledge learned from the tumorigenesis of other tumors [15–19, 42] and the *huMETCAM*-mediated progression of melanoma [43–45] and angiogenesis [2, 46–51], we may be able to find some common clues to begin understanding its mechanisms.

**Figure 16:**
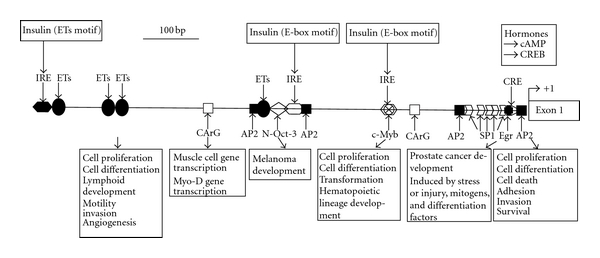
The promoter of the *huMETCAM* gene. The locations of various transcriptionally regulatory elements are shown in the 900 bp promoter region of the *huMETCAM* gene. The possible function of each element is also indicated. The promoter contains four AP-2 (activator protein-2, GCCNNNGGC), one CRE (cAMP response element, TGACGTCA), one Egr (early growth response element, CCCTG), five SP-1 (CCGCCC), two CArG (CC(A/t)6GG), three IRE (two insulin responsive elements with E-box motif, CANNTG, and one with Ets motif, ACGGAT), one c-Myb (coincided with one IRE with E-box motif, CACCTG), one N-Oct-3 (or brn-2, GCCTGAAT), and four Ets elements (GGAA).

 First, the transcriptional expression of *METCAM* gene may be regulated by *PKA/CREB *(cAMP-responsive element binding protein), *AP-2*α** [44, 45], and other transcription factors, such as *SP-1*, *c-Myb*, *N-Oct2*, *ETs*, *CArG*, *Egr-1,* and transcription factors binding to insulin-response elements, as shown in Figure 16 [7].

Among these potential regulators, it is well documented that the *AP*-*2*α** transcription factor plays a crucial tumor suppressor role in the progression of melanoma, prostate, and breast cancer [45]. It has been shown that *PKA/CREB* plays a positive role in the progression of melanoma, and perhaps also applicable to breast cancer and prostate cancer, by inhibiting the expression of AP-*2*α**and increasing the expression of *METCAM* [45]. However, the expression level of *AP-2*α**in other cancers has not been explored. The roles of other transcription regulators, tissue-specific enhancers and repressors, epigenetic control, and control at the level of chromatin remodeling of the gene have still yet to be investigated [7].

 Second, since the cytoplasmic tail of *METCAM* contains consensus sequences potentially to be phosphorylated by *PKA*, *PKC*, and *CK2*, it may manifest its functions by crosstalk with various signaling pathways mediated by these protein kinases [7]. For example, *METCAM *expression in melanoma cells is reciprocally regulated by *AKT*, in which *AKT *up-regulates the level *METCAM* and overexpression of *METCAM* activates endogenous *AKT*, which in turn inhibits apoptosis and increases survival ability [43]. However, it is not clear if a similar mechanism is also used in breast, prostate, and other cancers. Also, the detailed mechanism of how *AKT *up-regulates the expression of *METCAM* has not been worked out. *PKA*, *PKC*, and *CK2* may phosphorylate the cytoplasmic tail of *METCAM*, which then facilitates its interaction with *FAK,* thus promoting cytoskeleton remodeling. Alternatively, after phosphorylation of its cytoplasmic tail by these protein kinases, *METCAM* may interact with the downstream effectors of *Ras*, activating *ERK *and *JNK,* which in turn may transcriptionally activate the expression of *AKT* or other genes that promote the proliferation and angiogenesis of tumor cells. Though *METCAM *has not been shown to be a substrate of *CK2,* which has been shown to phosphorylate other *CAMs*, such as *CD44*, *E-cadherin*, *L1-CAM*, and *vitronectin*, it is also likely that *CK2* may be able to phosphorylate *METCAM* [46] and link it to *AKT* to affect the proliferation, survival, and other tumorigenesis-related functions of tumor cells [47].

 Third, after the engagement of *METCAM* with the ligand(s) or extracellular matrix, it may transmit the outside-in signals into tumor cells by activating *FAK* and the downstream-signaling components, promoting cytoskeleton remodeling and increasing tumor cell motility and invasiveness [2, 7].

 Fourth, from what we know about the roles of other *CAM*s in the progression of other tumors [15–19, 42], it is logical to postulate that *METCAM *may affect cancer cell progression by crosstalk with signaling pathways that affect apoptosis, survival and proliferation, and angiogenesis of tumor cells [7, 18, 42]. Thus, *METCAM* may affect tumorigenesis and metastasis by altering the expression of various indexes in apoptosis, survival signaling, proliferation signaling, and angiogenesis. To support this notion, we have found that *METCAM* promotes the progression of prostate cancer cells by rendering the cells with increased proliferative ability by elevating levels of *Ki67* and *PCNA*, with increased survival ability by elevating the level of phosphorylated *AKT*, and with increased angiogenic ability by elevating levels of *VEGF*, *VEGFR2,* and *CD31 *[35]; but it has no effect on the process of apoptosis. In contrast to this, *METCAM *promotes the progression of melanoma cells differently by preventing the apoptosis of melanoma cells [47] and reciprocally affecting the expression of a survival index, phospho-*AKT *[43]. Further systematic studies by using specific RNAis to knockdown the downstream effectors one-by-one in the *METCAM*-expressing clones may be necessary to further understand this aspect of mechanism.

 Fifth, *METCAM* may mediate hematogenous spreading of melanoma cells, which had been implicated by its expression in endothelial cells, as well as in malignant melanoma cells [48], further shown to be present in the junctions of endothelial cells [49, 50] and essential for tumor angiogenesis in at least three tumor cell lines [51] and human prostate cancer *LNCaP* cells [52]. It is highly likely that *METCAM* expression may promote hematogenous spreading of prostate cancer cells, similar to melanoma cells [49]. Similar mechanisms may also be used for the *METCAM*-mediated hematogenous spreading of breast cancer and osteosarcoma cells. However, it is not known if *METCAM* also plays a role in the lymphatic spread of cancer cells. Recent results from one group showed that *METCAM* is one of the lymphatic metastasis-associated genes, which is upregulated in malignant mouse hepatocarcinoma [53]; suggesting that *METCAM* may also play a role in promoting lymphatic metastasis of cancer cells. However, the details of how *METCAM *mediates hematogenous or lymphatic spreading of cancer cells have still yet to be investigated. Labeling the cells with viable dyes and following the process in real time by using a newly developed nonintruding, but highly photo-penetrating imaging method of photoacoustic tomography (PAT) [54, 55] may be useful for monitoring each step in the *METCAM*-mediated progression. For the *METCAM*-mediated dynamic spreading of melanoma cells *in vivo*, the PAT imaging method coupled with using hairless syngeneic mouse animal models [56] should reveal more clearly the process in real time.

 Sixth, *METCAM* has been shown to express in normal mesenchymal cells (smooth muscle, endothelium, and Schwann cells) in the tissue stroma and be a marker for the mesenchymal stem cells [57], *METCAM *may play an important role in regulating tumor dormancy or awakening, driving or preventing cancer cells to premetastatic niche, and formatting a microenvironment for favorable or unfavorable tumor growth in secondary sites.

 Seventh, *METCAM* may affect the progression of cancer cells by interactions with the host immune system, which, however, has been shown to have a paradoxical role in tumor progression [58]. Recently, one group has shown that a subset of host B lymphocytes may control melanoma metastasis through METCAM-dependent interaction [59]. On the other hand, it is highly likely that the tumor suppression effect of *METCAM* expression in melanoma *K1735-9* subline may be due to the interaction of *METCAM*-expressing cells with the host immune defense system in the immunocompetent syngeneic *C3H* brown mouse, since the intrinsic motility and invasiveness of mouse melanoma *K1735-9* was increased by the *METCAM* expression [22, 23]. For example, the surface *METCAM* expressed in this particular melanoma cell line may have a homophilic interaction with the *NK* cells, which also express *METCAM* and enhance cytotoxic functions of *NK* cells [60]. This hypothesis should be testable by studying the METCAM-mediated metastasis of METCAM-expressing K1735-9 cells in various genetically altered mice with a knockout of *CD4+ T* cells, *CD8+ T* cells, or *NK *cells, or mice with a combined knockout of these immune cells.

 Eighth, malignant progression of cancer cells has been shown to associate with an abnormal glycosylation, resulting in expression of altered carbohydrate determinants [61]. Thus, the glycosylated status of *METCAM* in different cancer types may be different from normal cells, thus manifesting positive or negative effect on the progression of different cancer types. This aspect of the METCAM-mediated cancer progression has not been well studied, but is especially intriguing since METCAM possesses six conserved N-glycosylation sites in the extracellular domain [7, 8].

We should always keep in mind that mechanisms of *METCAM*-mediated cancer progression may be slightly different in different cancer cells due to their different intrinsic properties, which provides different cofactors and/or different ligand(s) that either positively or negatively regulate the *METCAM*-mediated tumorigenesis and metastasis. To further understand the role of *METCAM* in these processes, it is essential to diligently identify the cofactors and the *METCAM*-cognate heterophilic ligand(s), which modulate the biological functions of *METCAM*. The endeavor in this direction appears to be promising from our preliminary attempts that we may have successfully found a possible candidate of METCAM's heterophilic ligand in *METCAM*-expressing human prostate cancer cells [7].

 Mechanisms of *METCAM-*mediated negative role in the progression of some cancer cells have not been studied at all. Does *METCAM* in some cancers behave like E-cadherin, which always plays a negative role in the tumorigenesis and metastasis of most epithelial cancer cells [18]? But even *E-cadherin* may function differently in different cancer cells. For example, its expression is temporally different and correlates with different stages during the progression of ovarian cancer [62]: *E-cadherin* is not expressed in the ovarian surface epithelial cells, expressed in premalignant lesions and in well-differentiated tumors, and finally not expressed in late-stage invasive tumors [62]. Likewise, *METCAM* may express and function normally in the normal nasopharyngeal epithelium, transiently reduce its expression and lose its function during the development of nasopharyngeal carcinoma, resume its expression, and function in the invasion stage of the cancer. Alternatively, METCAM may behave differently from *E-cadherin* by being modulated by different cofactors or ligands, which are expressed at different stages of the cancer. The tumor suppressor role of *METCAM* in ovarian cancer cells may not be due to the altered intrinsic properties of the cancer cells, since the intrinsic motility and invasiveness of human ovarian cancer BG-1 and *SK-OV-3 *cells was not affected by the *METCAM* expression [34, 35]. Our preliminary results appear to suggest a special mechanism that a soluble form of *METCAM*, which is produced by *MMP*s in the METCAM-expressing cells, may mediate the suppressive effect in ovarian cancer cells, similar to the production of a soluble form of *P-cadherin* by the induced MMPs in breast cancer cells, which then dictates, instead of suppresses, the aggressive behavior of the breast cancer cells [63].

## 5. Conclusions and Clinical Applications

*METCAM *may have a key positive function in the progression of angiosarcoma, breast cancer, osteosarcoma, prostate cancer, and most melanoma cell lines. On the other hand, it may also have a key function in suppressing the progression of a few melanoma cell lines, ovarian cancer, haemangioma, and other cancers. To further understand its mechanisms in these processes, it is crucial to define its functional domains, identify its cognate ligand(s) and cofactor regulators, and study its crosstalk with members of various signaling pathways [7]. These model systems may be useful for real-time observation of the dynamic process of cancer progression by using a nonintrusive and high photo-penetrating imaging system, such as the newly developed photoacoustic tomography (*PAT)*, to further understanding the process in mouse models [54, 55]. The knowledge gained would also be useful for designing effective means to decrease, or even to block the metastatic potential of these cancers. Along these lines, a preclinical trial of using *doxazosin*, an *α*1-adrenergic antagonist that has been used to treat the *BPH *patients, has been shown to be able to suppress prostate cancer metastasis in the *TRAMP* mouse model [64]. Furthermore, preclinical trials using a fully humanized anti-*METCAM* antibody against melanoma growth and metastasis [65, 66] and using a mouse anti-*METCAM* monoclonal antibody against angiogenesis and tumor growth of hepatocarcinoma, leiomyosarcoma, and pancreatic cancer [51] have been successfully demonstrated. Alternatively, small soluble peptides derived from *METCAM* may also be useful for blocking the tumor formation and tumor angiogenesis [52, 67, 68]. The attachment of these reagents to nanoparticles may be another alternative for therapeutic use [69].

## Figures and Tables

**Table 1:** The role of *METCAM* in the tumorigenesis and metastasis of various cancer cells.

Cancer cells	Tumorigenesis	Metastasis	References
Clinical prostate cancer and human prostate cancer cell lines	Increasing	Increasing (effect is in the early stage of initiation)	[7, 8, 12, 13, 35, 36]
Mouse prostate carcinomas in TRAMP mice	Increasing	Increasing	[41]
Clinical melanoma and human melanoma cell lines	No effect	Increasing (effect is in the late stages)	[3, 20]
Mouse melanoma cell line of K1735 subline number 3 and 10s	No effect or slight suppression	Increasing (effect is in the late stages)	[9, 21]
Mouse melanoma cell line K1735 subline number 9	Suppression	Suppression	[22, 23] and Wu et al. unpublished results
Angiosarcoma	Increasing	Not determined	[7, 37] and Wu et al. unpublished results
Human ovarian cancer cell lines BG-1 and SK-OV-3	Suppression	suppression	[33, 34]
Human osteosarcoma cell lines	Not determined	Increasing	[39]
Human breast cancer cell line MCF-7	Suppression	Not determined	[24]
Human breast cancer cell lines MCF-7 and SK-BR-3	Increasing	Not determined	[30, 31]
Haemangiomas	Suppression?	Not determined	[38]
Nasopharyngeal carcinoma	Suppression?	Not determined	[7, 11]
